# Stability of the V and Co atomic wires: a first-principles study

**DOI:** 10.1039/c8ra07895e

**Published:** 2018-12-12

**Authors:** Shu-Lan Liu, Bao-Ru Wang, Qing-Min Ma, Zun Xie

**Affiliations:** Department of Physics, Tangshan Normal University Tangshan 063000 Hebei China; Department of Physics, Hebei Advanced Thin Film Laboratory, Hebei Normal University Shijiazhuang 050024 Hebei China zxie@hebtu.edu.cn; College of Science, Hebei University of Science and Technology Shijiazhuang 050018 Hebei China

## Abstract

We employ density-functional theory calculations plus pseudopotentials with the projector-augmented wave method to investigate the structural stability and electromagnetic characteristics of two infinite atomic wires made of vanadium (V) and cobalt (Co). We identify five stable V atomic wires and four stable Co atomic wires. The H structure of the V atomic wire shows semiconductor characteristics, and the other four structures show metallic properties. None of the V chains has magnetism. On the other hand, the four stable Co atomic wires have metal properties. The dimerized Co atomic chain is shown to be ferromagnetic with a maximum spin magnetic moment.

## Abstract 2

We employ DFT calculations with the PAW method to investigate the structural stability and electromagnetic characteristics of two infinite atomic wires made of vanadium (V) and cobalt (Co).

## Graphical abstract

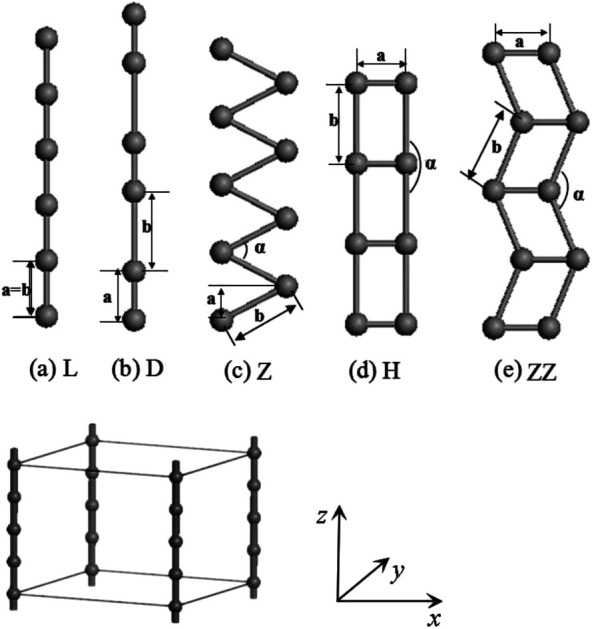

## 1. Introduction

Following the successful preparation of chains of four gold atoms,^1,2^ significant interest has been shown in other one-dimensional (1D) metal atoms. Theoretical studies^3–13^ that have been carried out so far have led to the conclusion that infinite metal nanowires are not linear (L) chains but rather, have only a quasi 1D geometry. For instance, Sanchez-Portal *et al.* calculated the properties of Au, Cu, Ca and K atomic chains^3,11^ using first-principles calculations and found that the wires prefer a planar zigzag (Z) structure with a bond angle of 60°. Sanchez-Portal *et al.* also suggested that Al atoms, having 3s^2^3p^1^ valence, form a ladder (H) structure^8^ comprised of two parallel linear chains that are close together. In short segments consisting of only a finite number of Au atoms, Häkkinen *et al.* found a stable, dimerized (D) linear chain through ab initio simulations.^13^ Using first-principles calculations based on density-functional theory (DFT), Swapan K. Pati *et al.*^14^ have demonstrated that the 1D infinite linear chain of vanadium atoms, which we refer to hereafter as a V-chain, prefers a dimerized form but the V–benzene multi-decker sandwich complex does not. The structural stability, electronic, and magnetic properties of the Ag-HMO system have been studied and it is found that the structure with linear Ag chains in the non-magnetic state is unstable.^15^

Generally, early 3d TM atoms prefer to form metal–benzene multi-decker compounds^16,17^ and met-cars,^18,19^ while late 3d TM atoms can form rice-ball structures^20,21^ such as those that are often used as catalytic particles in the production of single-wall carbon nanotubes.^22^ It is thus clear that the bonding characters can be very different in the early and late TM nano-materials. Compared with noble^3–10,13^ and alkali metal wires,^11,12,23^ the investigation of TM atomic wires^24–30^ is not yet sufficiently developed. S. Ciraci employed first-principles ultrasoft pseudopotential plane-wave calculations to investigate 3d TM atomic chains, including L and Z shapes. They found that the dimerization is obvious in nonmagnetic V chains but the linear Co chain does not favor a dimerized configuration.^29^ The unfilled d orbitals of the transition metals make the TM atomic wires much easy to magnetize,^31^ and it has been predicted that Ru, Rh, Pd, Os, Co and Pt atomic wires may also have magnetic ground states caused by the quantum confinement of electrons.^29,32–35^ In addition, it is known that TM atomic chains adsorbed onto carbon nanotubes or Si nanowires can exhibit high spin-polarity and magnetism.^36,37^ In fact, in spin electronics, the thinnest magnetic chains may be used for transporting spin-polarized currents.^38^ Therefore electron spin polarization may lower the total energy and improve the magnetism for specific TM chains.

Because both the electronic structure and magnetism of nanowires have close relationships with the geometry, many of the most basic problems regarding the properties of nanowires involve the structural stability of these nanowires. Vanadium and cobalt are, respectively, early and late 3d TM atom, with the 3d electron number of the Co atom being greater by four than that of the V atom. In order to understand the effect of different valence states on the stability, we have therefore systematically investigated infinite atomic wires made of V and Co, using the projector augmented wave (PAW) method^39,40^ based on DFT. For V wires, we have identified a new stable structure with a double zigzag (ZZ) shape, and for Co wires, we have found a new magnetic ground state with a ladder (H) shape. Both these studies confirm that the stability is related to the outmost valence electrons.

## 2. Computation details

The calculations of geometry optimizations and electronic structures have been carried out using density-functional theory (DFT) plus pseudopotentials with the projector-augmented wave (PAW) method as implemented in the Vienna ab initio simulation package.^41,42^ The exchange–correlation potential has been approximated by generalized gradient approximation (GGA).^43,44^ The cutoff energies of the plane wave basis were 200 eV and 300 eV, respectively for the V and Co wires. These values ensure that the change of total energy is within 1 meV per atom. The Monkhorst–Pack scheme with (1 × 1 × 40) *k* mesh points was adopted for the integration across the Brillouin zone, which further limits the error in the total energy to within 0.001–0.01 eV. Both spin-unpolarized and spin-polarized GGA calculations were carried out.

**Fig. 1:**
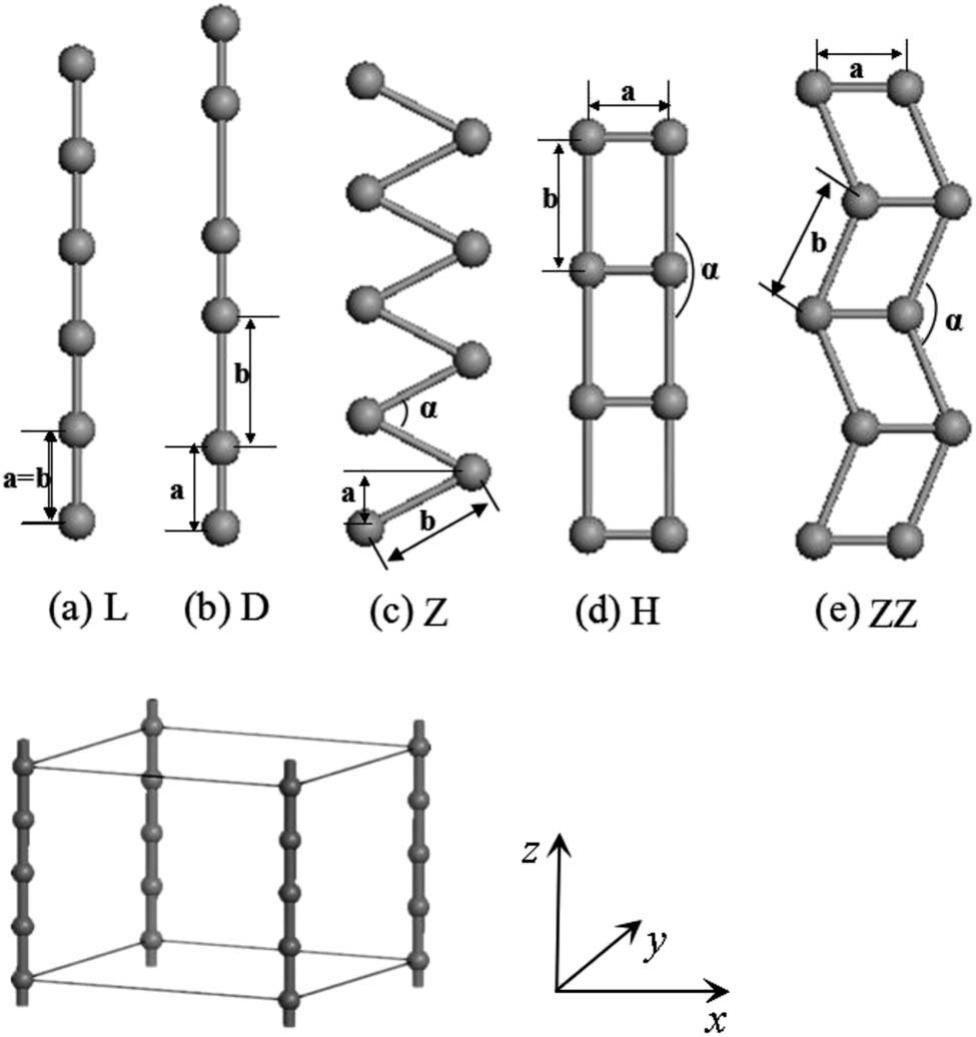
Five structures of atomic wires. (a) L, (b) D, (c) Z, (d) H and (e) ZZ show the linear, dimerization, zigzag, ladder and double-zigzag wire, respectively. The parameters *a*, *b* indicate the distances between two atoms in (a)–(e), and *α* is the bond angle. The supercell geometry and coordinates used in the calculations are also given.

Infinite periodic L and H chains and especially their distorted D and Z structures as well as ZZ structures have been thoroughly investigated (see Fig. 1). As shown in Fig. 1, the wire was treated as part of a supercell with the distance between wires set to 15 Å, which is large enough to ignore the interactions between the wires. We took the *z* axis to lie along the chain with the planar Z, ZZ and H structures located in *xz* plane. The fully-relaxed structures were obtained by requiring that the Hellmann–Feynman (H–F) forces on each atom be less than 0.001 eV Å^−1^.

As a test, we performed calculations on the body-centered cubic (BCC) bulk vanadium and hexagonal close-packed (HCP) bulk cobalt. The equilibrium lattice constants and cohesive energies (*E*_c_) are listed in Tables 1 and 2, and are in good agreement with the experimental results.^45^ The cohesive energy of the system is defined as
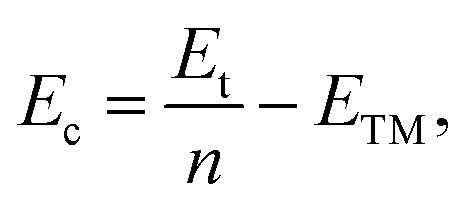
where *E*_t_ is the total energy of the supercell, *n* is the number of atoms in the supercell, and *E*_TM_ is the energy of a single transition metal atom V (Co).

**Table 1:** Calculated results for the V chains and BCC bulk, including the lattice constants *a* and *b*, the bond angle *α*, cohesive energy *E*_c_ and atomic coordination number CN

Structure	*a* (Å)	*b* (Å)	*α* (°)	*E*_c_ (eV per atom)	CN
L	1.91	1.91	180°	−1.75	2
D	1.65	2.70	180°	−2.26	2
Z	1.42	1.46	88°	−3.29	4
H	1.51	2.74	180°	−2.62	3
ZZ	1.81	2.42	130°	−3.04	3
BCC bulk	3.03			−5.39	8
BCC bulk (expt.)	3.03			−5.33	8

**Table 2:** Spin un-polarized results Co atomic wires and the HCP bulk, including lattice constants *a* and *b*, bond angle *α*, cohesive energy *E*_c_ and the coordination number CN

Structure	*a* (Å)	*b* (Å)	*α* (°)	*E*_c_ (eV per atom)	CN
L	2.07	2.07	180°	−2.12	2
D	1.90	2.25	180°	−2.04	2
Z	1.12	1.99	59°	−2.90	4
H	2.12	2.62	180°	−3.03	5
HCP bulk	2.50	4.10		−5.47	12
HCP bulk (expt.)	2.51	4.07		−4.39	12

## 3. Results and discussions

### 3.1 V atomic wires

**Fig. 2:**
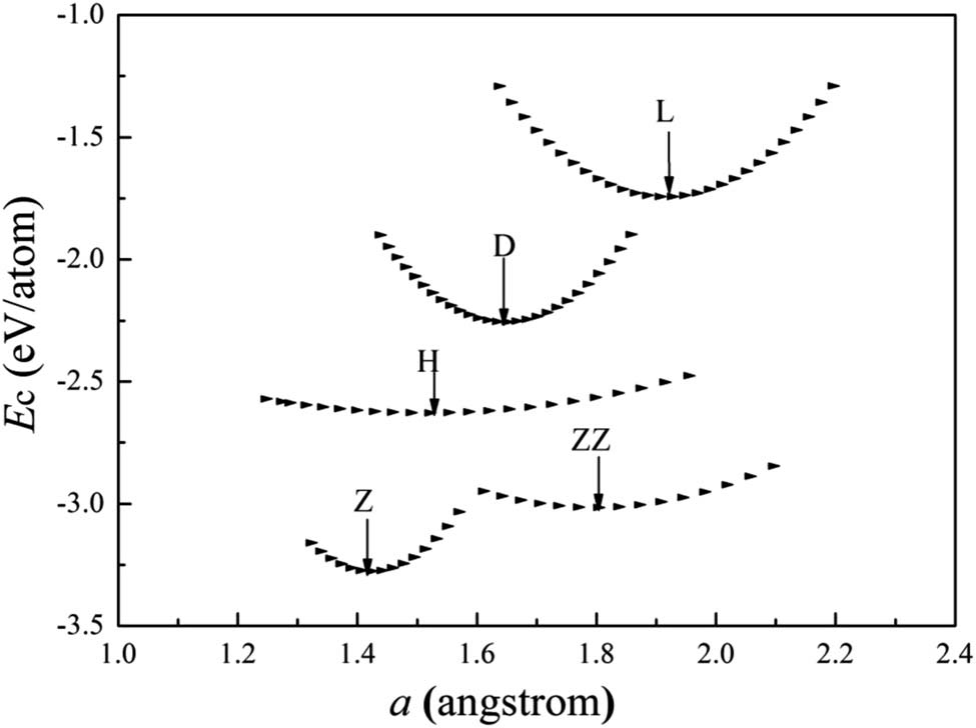
Calculated cohesive energies of V atomic wires as a function of the structural parameter “*a*”. The local minimum point of each curve is denoted by an arrow.

The cohesive energies of V wires as a function of the structural parameter are shown in Fig. 2, and the optimized structural parameters, cohesive energies of the wires and the BCC bulk V are listed in Table 1.

The present calculations indicate that vanadium can form five kinds of stable wires: L, D, Z, H and ZZ structures. The most stable geometry, *i.e.*, with the lowest cohesive energy, is the Z structure and its energy minimum occurs at (1.42 Å, −3.29 eV) as shown in Fig. 2. This is the most stable structure and has been referred to as the Z configuration by S. Ciraci.^29^ The cohesive energy of the H structure (in Fig. 1(d)) is 0.88 eV per atom lower than that of the L structure. It is noted that the distance “*a*” between chains is much shorter than the distance “*b*” between atoms along the chains, which indicates that the bonding between two nearest chains of the H structure is stronger than that along the chain in the *z* direction. Furthermore, allowing the distortion of the bond angle “*α*” from the linear H configuration to 130°, a new stable ZZ wire has been identified for the first time using a first-principles calculation. This distortion from H to ZZ lowers the cohesive energy by 0.41 eV per atom.

The cohesive energy of the ideal linear L chain is also lowered, by 0.51 eV per atom, *i.e.* the linear V chain prefers to take on the D form in which the atoms are unequally spaced.^14,29^ The most stable geometry of the D chain occurs with *a* = 1.65 Å (which is 0.26 Å shorter than that of the ideal L chain) and *b* = 2.70 Å (as shown in Fig. 2). The above analysis of the structural stability shows that the structures with higher symmetry are unstable and that distortions are bound to appear for a wire composed of V atoms with only three valence electrons.

From Table 1, we see that the cohesive energy is related to the coordination number (CN): the greater the CN, the lower the cohesive energy. This is in accord with what has been seen with Ti and Zr chains.^28,30^ The BCC bulk has the highest coordination number and the lowest cohesive energy, but the bond length in the quasi 1D wire is much shorter than that of the bulk, so it is interesting to compare the cohesive energy per coordination. For example, in the Z chain and BCC bulk, there are four and eight nearest neighbors, respectively. The cohesive energies divided by the CN are then 0.875 eV and 0.67 eV for the Z chain and BCC bulk, respectively. Apparently, the bonding in the Z chain is stronger than that in the bulk, which agrees with the results previously obtained for Al^12^ and Nb^8^ atomic wires.

**Fig. 3:**
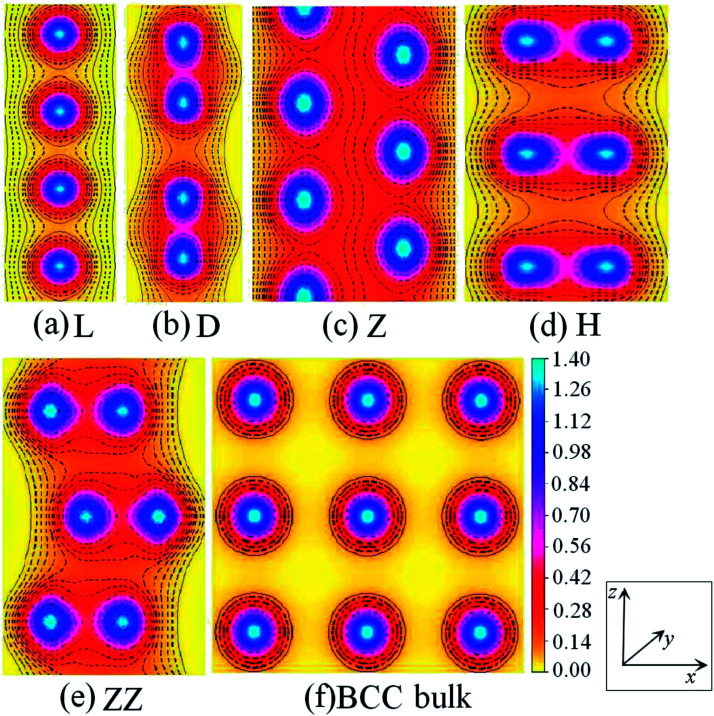
Charge-density contour plots for V atomic wires in *xz* plane: (a) L, (b) D, (c) Z, (d) H and (e) ZZ structure and for (f) the (111) plane of BCC bulk. The increment is 0.1 electrons per Å^3^.

The charge-density shown in Fig. 3 plot provides further insight into the stability and the bonding character. Fig. 3 shows the charge-density contour plots of V atomic wires in the L, D, Z, H and ZZ configurations, as well as for the (111) surface of BCC bulk vanadium. A metallic distribution of charge density is clearly seen in bulk (Fig. 3(f)) and in the L chain. The charge density distribution of the V atomic wires is different from that of the Nb^8^ atomic chain. In the case of Nb, the charge density distribution has no obvious directionality and appears spherical similar to the corresponding distribution. Weak directionality can be seen in the Z chain (Fig. 3(c)). However, more strongly directional ‘‘covalent’’ bonds are exhibited in the D, H and ZZ structures (Fig. 3(b), (d) and (e)). In the ZZ and H structures, the bonding between two adjacent chains is obviously stronger than it is along the *z* axis, and indeed, the lattice parameters “*a*” (1.81 Å for ZZ and 1.51 Å for H) in the *x* direction are clearly shorter than that along the chain direction (*b* = 2.42 Å and *b* = 2.74 Å, for ZZ and H respectively).

**Fig. 4:**
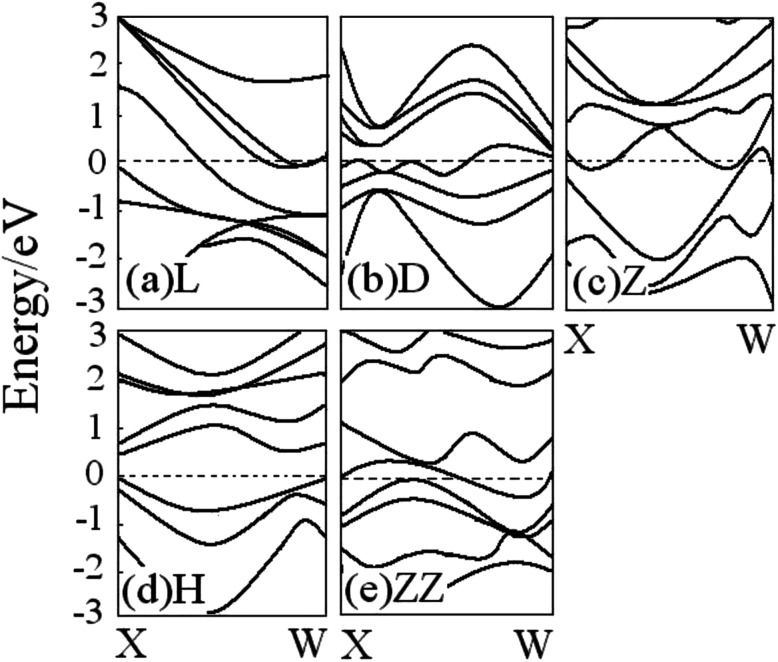
Energy band structures for all V atomic wires from spin-unpolarized calculations ((a) L, (b) D, (c) Z, (d) H, (e) ZZ). The Fermi energy is located at the zero of each band (dotted line).

The geometry of the wire clearly determines its electronic structure. Fig. 4 shows the energy band structure for all V atomic wires mentioned above. The V atom contains s and d valence electrons, and the band structure of the V wire is therefore more complicated than that of the Au and Al wires discussed elsewhere. For the various V wires, there is diversity near the Fermi level. Both the L and Z wires show metallic properties with no band gap, and there are more electronic bands crossing the Fermi level for the Z structure than L structure, which implies that the Z structure has the stronger conductivity. On the contrary, there is no energy band passing through the Fermi level in the H structure (Fig. 4(d)), and the band gap is about 0.52 eV, which indicates semiconductor characteristics. The energy band structure becomes more metallic from the H to ZZ (Fig. 4(e)). Likewise, the linear metallic V chain (Fig. 4(a)) becomes more metallic upon dimerization (Fig. 4(b)), which is consistent with Ciraci's conclusion.^29^ These interesting transitions show the dependence of the bonding character and the electronic structure on the geometric structure of the wire.

**Fig. 5:**
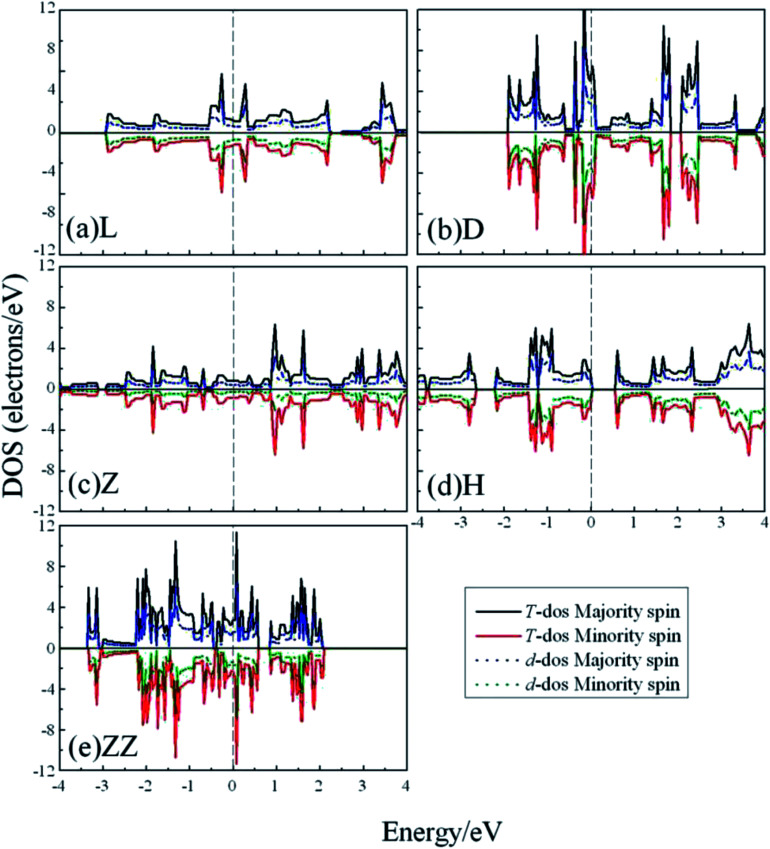
The total DOS and d-projected PDOS for various V atomic wires from spin-polarized calculations. The dashed line indicates the Fermi level which is shifted to zero.

To explore whether the V atomic wires are magnetic, spin-polarization calculations were also carried out. The total density of states (TDOS) and the d-projected partial density of states (PDOS) are plotted in Fig. 5. The PDOS show that the 3d electrons of V atomic wires make the largest contribution to the electronic density near the Fermi level. The curves for the majority-spin and minority-spin are symmetric and there is no relative migration, which indicates that none of the V chains exhibits high spin-polarity and thus has magnetism.

### 3.2 Co atomic wires

**Fig. 6:**
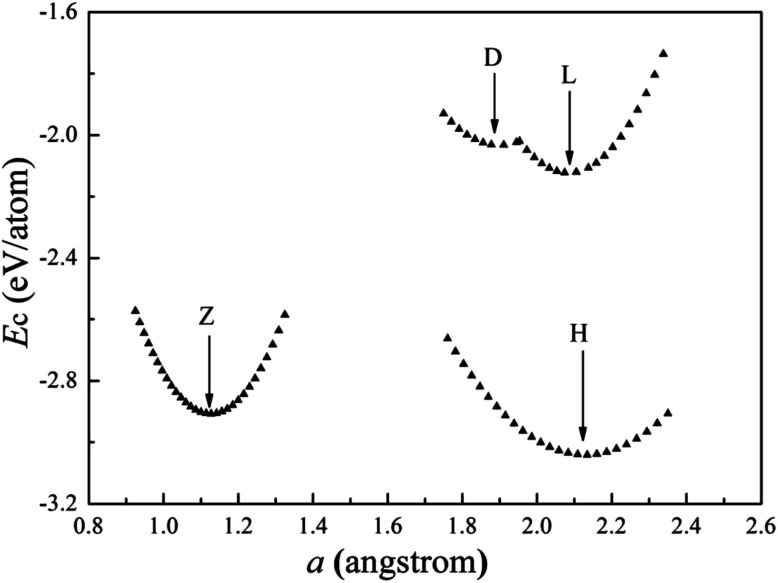
Calculated cohesive energies of Co atomic wires as a function of the structural parameter “*a*”. The local minimum of each curve is indicated by an arrow.

The cohesive energies of the cobalt wires as a function of the structural parameter “*a*” calculated for the spin un-polarization case are shown in Fig. 6. Table 2 lists the structure parameters for the Co atomic wires and the HCP bulk.

We have found four stable Co atomic wires. Our Z chain is the ground state of the Co atomic wire described in ref. 29, and there referred to as the dimerized zigzag. Here, the Z chain is only the second low-lying state. Among all chains obtained here, the H chain has the lowest cohesive energy and the largest coordination number 5. The cohesive energy is 0.13 eV per atom lower than that of the Z wire. As in the H structure of the V wire, the distance “*a*” between Co atoms in neighboring chains is shorter than the distance “*b*” between atoms in a chain. The difference is that the bond length *b* in the H Co wire is shorter than that in the V wire, which leads to a larger CN in the Co wire (5 *vs.* 3). Unlike the V atomic wire, the ideal linear Co chain is more stable than the D chain. In fact, the cohesive energy of the D chain is 0.24 eV per atom higher than that of the L and 1.31 eV per atom higher than that of the H ground state. The ZZ configuration is unstable for the Co chain. In summary, it appears that the Co wire lowers its energy mainly by bonding between two adjacent L chains and increasing the CN. The present work supports the conclusion of ref. 29, that the distortion appears to depend on the number of 3d electrons. The 3d electron number of the late TM Co atom is much larger than that of the early TM V atom, so the ground states of Co and V atomic wires are different.

**Fig. 7:**
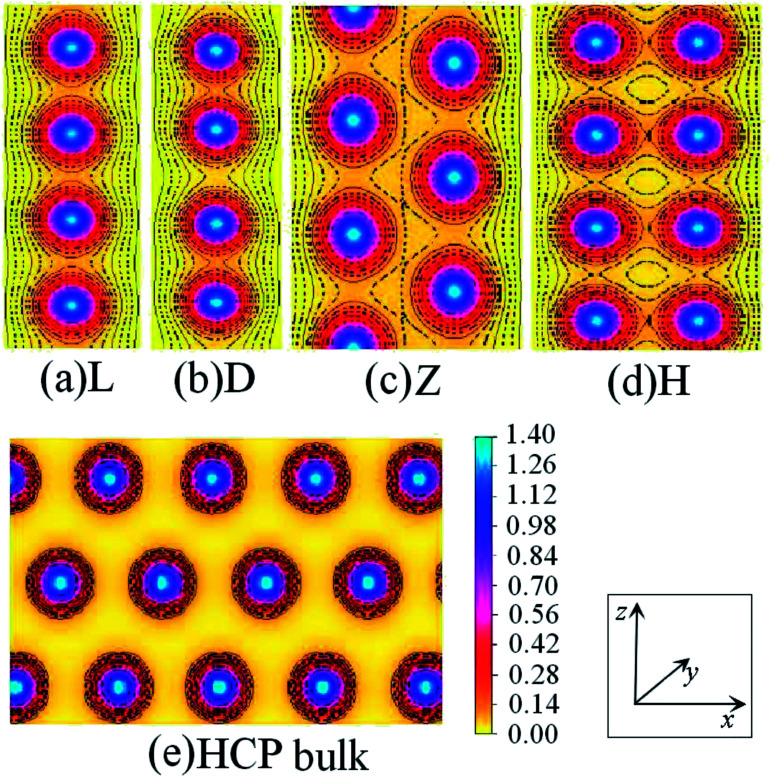
Charge-density contour plots of Co atomic wires in the *xz* plane: (a) L, (b) D, (c) Z and (d) H structure together with (e) the (0001) plane of HCP bulk. The increment is 0.1 electrons per Å^3^.

**Fig. 8:**
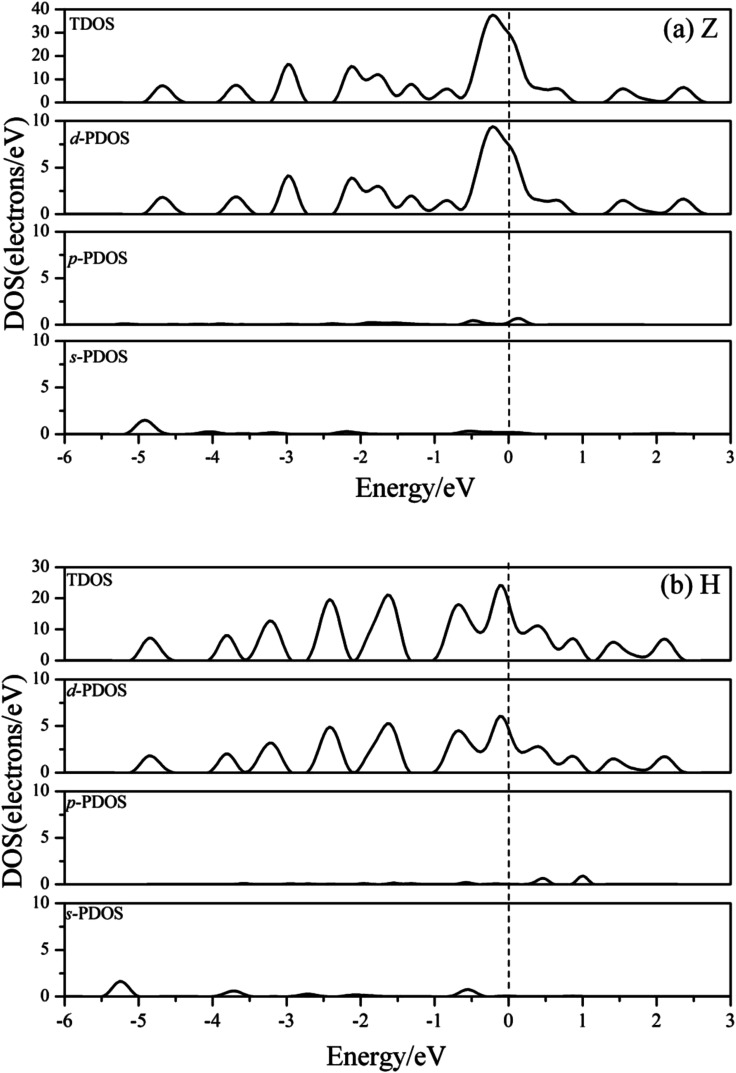
The TDOS and s-, p-, d-projected PDOS for the (a) Z and (b) H structure of Co atomic wires in spin-unpolarized calculations. The dashed line refers to the Fermi level which is shifted to zero.

Fig. 7 shows the charge density contours along the length of the Co atomic wires in the *xz* plane and in the (0001) plane of HCP bulk Co. The signatures of metallic bonding are obvious in all structures. That is to say, the charge distribution is spherical and there is no obvious directionality between the atoms. We also calculated the TDOS and PDOS in the spin un-polarization case, which also shows metallic bonding in all infinite Co wires. The TDOS and the s-, p-, and d-projected DOS for the Z and H structures are illustrated in Fig. 8(a) and (b). The discrete state densities and multimodal shapes suggest metal character, which is induced by the limitation to only two dimensions. Note that the 3d electrons play a dominant role near the Fermi level, whereas the contributions of the s and p electrons are negligible.

Cobalt is a magnetic element, so for Co atomic wires, we used the spin-polarized GGA to investigate the variation of cohesive energies in different magnetic states. Table 3 shows the structural parameters of the Co atomic wires in the ferromagnetic (FM), anti-ferromagnetic (AFM) and non-magnetic (NM) states.^15,46^ These results show that the L chain can exist only in the NM state. All of the other three kinds of wires prefer the FM state. The FM structure of the H chain appears to be the lowest energy structure for Co. Note that, the FM polarization reduced the cohesive energies by about 1 eV compared with the spin-unpolarized results for all of the structures in Table 2. The spin magnetic moment of the dimerized FM Co chain is the largest. From Table 3, it may also be seen that for the D chain, the lattice parameters, *a* and *b*, are 1.95 Å and 2.42 Å in the NM state, 0.06 Å and 0.07 Å less than that in FM state, respectively. This demonstrates that increasing the bond length favors magnetic polarization. It thus appears that the longer the bond length, the less the wave functions overlap, and the stronger is the magnetism. The last column in Table 3 shows that the magnetism tends to decline from the D structure to the Z and then to the H, which suggests that the higher the dimension, the larger the atomic CN, and the weaker the magnetism. In this case, the higher dimension and the larger CN cause broadening of the d electron state, which makes spin-splitting quite difficult and results in the magnetism becoming weak.

**Table 3:** Structural parameters of the Co atomic chains in different magnetic states, including lattice constants *a* and *b*, bond angle *α*, cohesive energy *E*_c_ and the magnetic moment MM

Structure	State	*a* (Å)	*b* (Å)	*α* (°)	*E*_c_ (eV per atom)	MM (*μ*_B_ per atom)
L	NM	2.07	2.07	180°	−2.97	—
D	NM	1.95	2.42	180°	−2.73	—
	FM	2.01	2.49	180°	−2.83	1.752
Z	NM	1.16	2.24	62°	−3.70	—
	FM	1.15	2.27	61°	−3.76	1.512
H	AFM	1.11	2.29	58°	−3.73	1.385
	NM	2.03	2.20	180°	−4.02	—
	FM	2.05	2.23	180°	−4.04	1.003

**Fig. 9:**
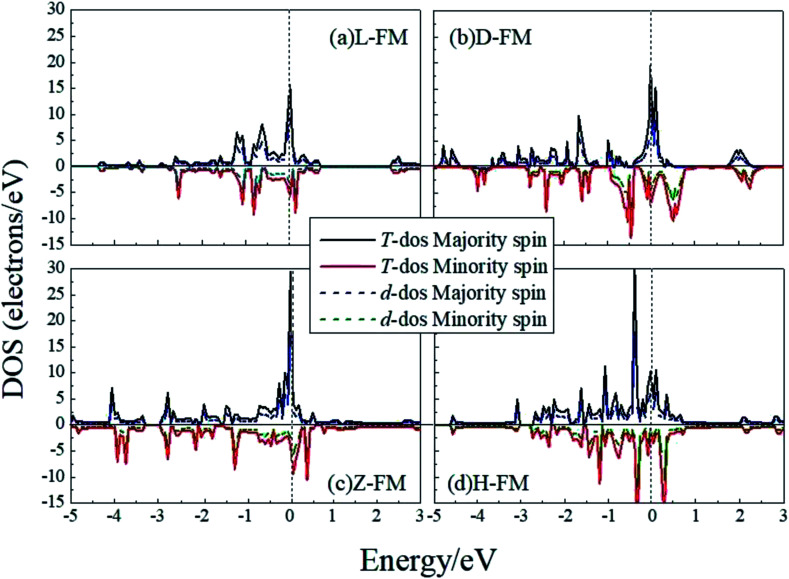
The total DOS and d-projected PDOS for the four kinds of Co atomic wires. The dashed line refers to the Fermi level which is shifted to zero.

In order to have a better understanding of the magnetic properties of the wires, we plot the TDOS and d-projected PDOS for the four kinds of Co atomic wires in Fig. 9. The DOS presents many peaks because of the two dimensional limitation. The relative displacements between the spin states are very obvious for the FM state of the four Co atomic wires, which indicate the generations of more spin parallel d electrons.

## 4. Conclusions

Infinite periodic linear (L) and ladder (H) structures of V and Co atomic wires, with emphasis on their distorted structures (D, Z and ZZ structures) have been investigated using the DFT calculations plus pseudopotentials with the PAW method. It was found that in each case, the structural stability of the atomic wire was affected strongly by the outmost valence electrons and that the bonding characteristics and the magnetism have a close relationship with the geometry. Specifically, we have identified five stable V atomic wires and four stable Co atomic wires here. For the V atomic wire, we identify a new stable ZZ structure. This wire is a distorted form of the L structure. The metallic L structure of V chain becomes more metallic with D structure.

Both the bonding characters and the magnetism have close relationships with the geometry. Bonding two L chains to form an H structure causes the energy of the L structure of Co atomic wire to decrease with the result that the preferred ground state is a magnetic H structure. The D structure has the largest spin magnetic moment of all the Co atomic wires investigated.

## Conflicts of interest

There are no conflicts to declare.

## Supplementary Material
